# Assessment of Phytoremediation Potential of* Chara vulgaris* to Treat Toxic Pollutants of Textile Effluent

**DOI:** 10.1155/2019/8351272

**Published:** 2019-02-03

**Authors:** Pooja Mahajan, Jyotsna Kaushal, Arun Upmanyu, Jasdev Bhatti

**Affiliations:** Department of Applied Sciences, Chitkara University, Rajpura 140401, India

## Abstract

Textile effluent released into water bodies is prone to be toxic for aquatic flora and fauna. In the present study, the phytoremediation potential of* Chara vulgaris *(*C. vulgaris) *is investigated for treatment of textile effluent. The highly concentrated and toxic textile effluent is diluted to different concentrations 10%, 25%, 50%, and 75% to check the accessibility of macroalgae to bear pollutant load of textile effluent. The toxicity of textile effluent is analysed by determining different water quality parameters, namely, pH, TDS, BOD, COD, and EC. The maximum reductions in TDS (68%), COD (78%), BOD (82%), and EC (86%) were found in the 10% concentrated textile effluent after 120 h of treatment. The highly concentrated textile effluent showed its toxic effect on macroalgae and it was found unable to show a remarkable change in water quality parameters of 75% and 100% textile effluent. The correlation coefficient values are determined using correlation matrix to identify the high correlation between different water quality parameters. The removal of toxic organic pollutants by* C. vulgaris* was confirmed by using UV-visible absorption spectra. Typical X-ray spectra recorded using EDXRF technique indicated the presence of heavy metals Cd in the dried sample of macroalgae after treatment which show its capability to remove toxic heavy metals from textile effluent. The reliability model has been proposed for treated textile effluents to identify percentage level of toxicity tolerance of waste water by macroalgae.

## 1. Introduction

Textile effluents released from the textile industry play a major role for polluting the water streams [1]. Textile effluents released into the environment not only cause water pollution, but also cause aesthetic problems as dye bearing effluents changed the colour of the water bodies which interferes with sunlight penetration and disturbs the aquatic ecosystem [2, 3]. Most of the dyes have carcinogenic action and also cause other skin problems like allergies, dermatitis, skin irritation, etc. Moreover, half-life time period of dyes is of several years, so their long time persistence in the environment causes accumulation in sediments, fishes, or other aquatic creatures [4]. Hence, it is mandatory to remove the contaminants from textile effluent before their discharge into the water bodies; otherwise it disrupts the aquatic environment.

A large number of conventional congenital methods, for instance, activated carbon adsorption, separation through the membrane, electrochemical coagulation, and ultrafiltration, are used by textile industry to treat its effluents [1]. All these methods have a potential to treat textile effluent to varying extent but high cost of these technologies puts the financial burden on industries and effluent without treatment results into hazardous water pollution. This problem leads to generation of other methods of textile effluent treatment [5]. Treatment of textile effluents by using solar driven plants through phytoremediation is a time tested, attractive, aesthetic, pleasant, eco-friendly, and cost-effective approach [6]. Researchers have identified the potential of* Typhonium flagelliforme, Phragmites australis, Ipomoea hederifolia, *and* Blumea malcolmii* for specific textile dyes removal [7–10]. Some decorative plants such as* Portulaca grandiflora, Aster amellus, Petunia grandiflora, Glandularia pulchella, Zinnia angustifolia, *and* Tagetes patula *have also shown their potential for degradation of textile dyes and effluents [11–15]. Chanshive et al. reported the use of various decorative plants* Tagetes patula, Aster amellus, Portulaca grandiflora,* and* Gaillardia grandiflora* for treatment of textile wastewater with constructed wetlands [16].

Among different categories of plants, aquatic macrophytes appear to be as more appropriate and successful for the treatment of effluent. The application of free floating aquatic species like* Eichhornia crassipes*,* Typha, Alternanthera philoxeroides,* etc. to treat textile effluent was proposed by researchers [17–19]. Recently, aquatic macrophytes such as* Typha domingensis, Fimbristylis dichotoma,* and* Ammannia baccifera* were used in various constructed wetlands for treatment of textile effluent [20, 21]. Among different aquatic species, in this study the potential for macroalgae* C. vulgaris* explored for treatment of textile effluent of different concentrations and treatment performance was measured via various water quality parameters.

*C. vulgaris* is also known as Musk grass categorized as submerged fresh water species and belongs to division Charophyta, order Charales, and family Characeae.* C. vulgaris* has wild distribution and possesses complex branching system with nodes and internodes which form beds inside water bodies up to a height of 1 metre [22]. The most salient feature of* C. vulgaris* is that it is able to develop even in high salt concentrations and it dominates over other submerged aquatic flora [23]. Fereshteh et al. reported absorption of heavy metal Arsenic by* C. vulgaris *[24]. Phytoremediation potential of* C. vulgaris* was earlier explored for a diazo dye Congo red [25]. The study revealed that* C. vulgaris* removes 95% dye from its aqueous solution within 24 h of experimentation. These finding were enough encouraging to select* C. vulgaris* for the treatment of textile effluent.

Thus, the focus of the present research is on progressive decolourization and preferential removal of contaminants of textile effluent by* C. vulgaris* which demonstrate its ability for the remediation of textile effluent. This study also provides a reliability model for successfully employing the phytoremediation system for textile effluent.

## 2. Materials and Methods

### 2.1. *C. vulgaris* Collection and Propagation

*C. vulgaris *was collected from the Botanical Garden of Panjab University, Chandigarh, India (an elevation of 321 m above sea level, 30.74°N 76.79°E) and was taken to the phytoremediation laboratory where it was cleaned for 6 hours under running tap water to remove attached contaminants. Thereafter, it was placed in cemented tank (0.76 m × 0.76 m) containing tap water and remained to grow, open to natural environment, for one month. Also, a Hoagland nutrient solution was fed into the tank for fast growth of algae [26]. Prior to experiments,* C. vulgaris* was again washed and treated with 0.2% HgCl_2_ for 2-3 minutes to avoid any type of contamination.

### 2.2. Experiment Set Up

**Figure 1:**
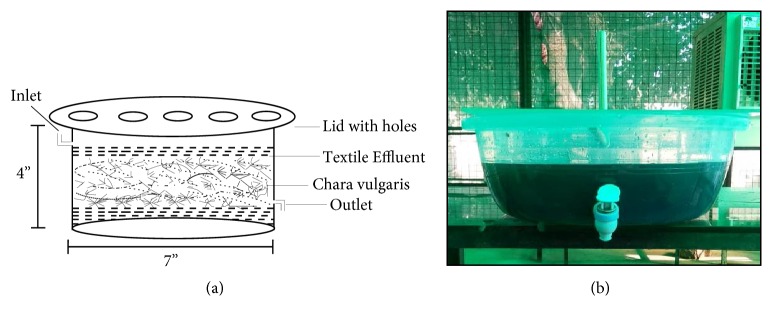
(a) Cross section view; (b) original image of Phytoreactor.

Textile effluent sample was obtained from the Effluent Treatment Plant (ETP) of a textile mill of Ludhiana city of Punjab (India) which is the hub of textile industry. The sample was collected after primary level of treatment using plastic containers and transported to laboratory. Six phytoremediation experiments were set up for remediation of the contaminants from the textile effluent. A phytoreactor was designed for each reaction by taking a cylindrical plastic tank of capacity 10 L. The cross section view and original image of phytoreactor are shown in Figure 1.

A plastic lid with holes of diameter 0.005 m was provided on top of the phytoreactor to protect the system from further contamination and to provide aeration to the system through natural environment. Through the inlet, 5 L of textile effluent was transferred into the phytoreactor. The first phytoreactor biotic control, which contained tap water along with macroalgae, was treated as biotic control, and other five phytoreactors containing untreated effluent of concentrations 10%, 25%, 50%, 75%, and 100% were evaluated for efficiency of treatment of textile effluent. No extra nutrients were added during phytoremediation treatment. The weight of macroalgae was optimized previously by performing experiments on a small scale. So, optimized average weight of 500 ± 0.50 g (wet weight was taken after placing it on filter paper) of* C. vulgaris* has been taken for each experiment [27]. To assess the phytoremediation potential for* C. vulgaris*, a retention time of 5 days (120 h) was provided for each experimental set up and experiments were performed in three batches.

### 2.3. Characterization of Textile Effluent

Different physiochemical parameters like pH (Glass electrode method: Jackson 1967), conductivity (Conductivity Meter: Jackson 1967), TDS (Filtration, Evaporation (103C) method: Standard Method APHA 2002), DO (Modified Winkler's Method: Standard Method APHA 2002), BOD (Modified Winkler's Method: Standard Method APHA 2002), and COD (Open Reflux Method: S.M. APHA 2002) of textile effluent were analysed from 0 to 120 h at intervals of every 24 h according to APHA [28]. Here, 0 h refers to the initial values of effluent parameters before introducing the* C. vulgaris* into the system. All the chemicals used for these characterizations were purchased from the Merck Company.

### 2.4. Analytical Techniques

The dried sample of macroalgae* C. vulgaris* was analysed for heavy metal by using radioisotope based EDXRF spectrometer. The targets were used in the form of thick pallets and the detector was attached to multichannel analyser (Canberra, Model S-100) to collect the X-ray spectra. UV-visible spectroscopy is used to analyse reduction in the toxicity level after treatment with* C. vulgaris*. For this purpose, 2 mL aliquots of 10% concentration of textile effluent were taken before and after treatment of 120 h. The supernatant was then analysed in a quartz cell of 1 cm optical length of an interval of 200–800 nm using a Systronic UV-2202 UV-visible spectrophotometer.

### 2.5. Phytotoxicity Studies

Phytotoxicity studies of textile effluent before and after treatment were also carried out at room temperature (30 ± 4°C). Six seeds of each* Pisum sativum* and* Phaseolus mungo* were placed in separate 5 mL solutions 50% untreated and treated textile effluent and with control tap water. The distilled water is used for watering the plants every day. After 10 days of sampling, length of shoot (plumule) and root (radical) were recorded and the following formula was used to calculate the Germination percentage [29]:

(1) Germination  %=Number  of  germinated  seedsTotal  number  of  seeds  sown×100

### 2.6. Statistical Analysis

To assess the phytoremediation potential for* C. vulgaris*, experiments were performed with textile effluent in multiples of three and average data was presented in the form of graphs and tables. Also, correlation studies were carried out to compare the different water quality parameters and evaluate significant relations of their r values. The correlation coefficient “r” was calculated with x and y being different physiochemical parameters variables using the equation [30].

(2) $$\begin{array}{c} r=\frac{\sum xy}{\sqrt{\sum x^{2}\times \sum y^{2}}} \end{array}$$

## 3. Results and Discussion

### 3.1. Characterization of Textile Effluent

The textile effluent was dark bluish black in colour with high pungent smell. The various physiochemical parameters of untreated textile effluent, namely, pH, EC, DO, TDS, BOD, and COD, were found to be very high in comparison to EPA acceptable limits [31] as shown in Table 1.

In preliminary experiments, growth of* C. vulgaris* was found to be retarded within 3 days of treatment due to noxious nature of chemical constituents used in textile industry. Hence, to check capacity of macroalgae to bear the pollutant load, it was decided to treat macroalgae by varying concentrations (10%, 25%, 50%, 75%, and 100%) of textile effluent. The amount of each plant and contact time for treatment process has been also optimized before conducting the experiment at pilot scale.

Temperature of textile effluent was recorded as 46°C at the time of collection, which was considered to be quite high. The optimum temperature required for the growth of macroalgae* C. vulgaris* ranges from 20°C to 35°C [32]. Hence, all phytoremediation experiments were carried out after one day of collection when temperature of the effluent came down to room temperature (30-34°C).

**Figure 2:**
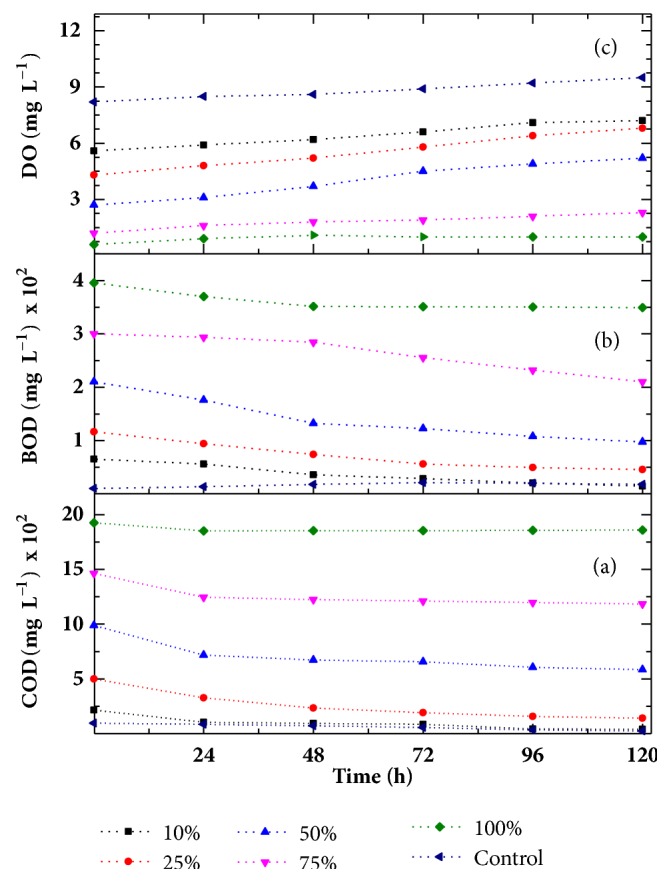
Variation in COD (a), BOD (b), and DO (c) as a function of time at different concentration.

**Figure 3:**
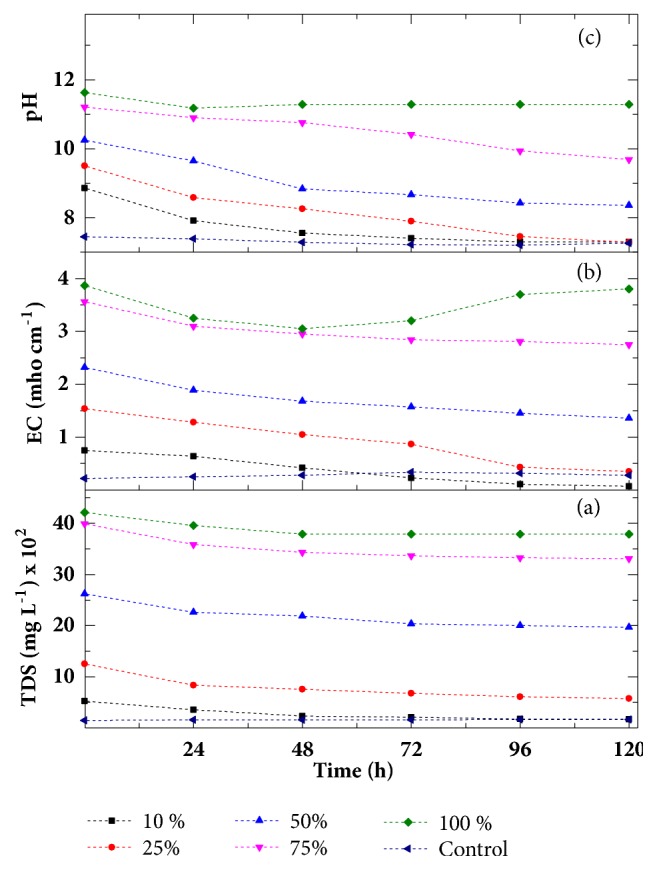
Variation in TDS (a), EC (b), and pH (c) as a function of time at different concentration.

In order to examine the efficiency of* C. vulgaris* for reducing contamination from effluent, various physiochemical parameters in different sets of concentration were analysed at regular interval of 24 h for 120 h and are given in Figures 2 and 3. The COD and BOD values of 100% textile effluent were found to be 1975 mg L^−1^ and 395 mg L^−1^, respectively. The high values of COD and BOD attributed to a number of dyes, detergents, oil, reducing agents, etc. utilized during dyeing and printing processes in textile mills. Also, the presence of dyes in water results in turbidity and imparts colour along with high BOD and COD levels. High BOD and COD levels introduce the hazardous pollution level in water bodies.

It has been clearly indicated in Figure 2 that in phytoremediation experiments ranging from 10% to 75% concentrations of textile effluent, BOD, and COD get reduced appreciably by* C. vulgaris* within 24 h of treatment. A remarkable reduction of 82% and 78%, respectively, in COD in Figure 2(a) and BOD in Figure 2(b) of 10% concentration was observed after 120 h which are the most important parameters of assessing pollution of water. These results are supported by earlier lab scale studies on phytoremediation of dye bearing effluent by using* Glandularia pulchella* which also have shown a remarkable reduction of 70% and 74% in COD and BOD within 60 h of treatment [5], Another phytoreactor was developed with* Portulaca grandiflora *which is found to be effective reduction of COD and BOD, by 59 and 38%, respectively, within 72 h of treatment [33]. The microalgae* Chlorella vulgaris have been *reported to reduce COD by 69% with the period of 14 days. The reduction in COD and BOD might be due to production of oxygen by photosynthetic plants which enhances the degradation of the organic matter present in the textile effluent and decreases its toxicity [34].

The collected effluent sample was completely devoid of Dissolved Oxygen content as DO value was found to be 0.62 that signified the unfavourable condition for the growth of aerobic organisms. In the present study, a fast progressive increase in DO values was found in 10%, 25%, and 50% after phytoremediation process of 120 h but, in both 75% and 100%, no significant change was observed in DO value as shown in Figure 2(c).

Another trend of increase in DO with the reduction in BOD and COD values was also observed in our study.* C. vulgaris* significantly reduces the BOD and COD levels of textile effluent. Similar trend was reported in literature by Tripathi and Shukla that, with increase in DO, there was significant reduction in BOD and COD. During treatment of wastewater with* Eichhornia crappies*, 70% increase in DO was observed with reduction of BOD and COD by 96.9% and 77.6%, respectively [35].

TDS and EC were closely related and found to follow almost the same trend of treatment performance during phytoremediation experiment. The reduction in TDS and EC values was observed within 120 h of treatment of all concentrations of textile effluent. The maximum reduction up to 68% and 86% observed TDS and EC, respectively, in 10% concentration after 120 h of treatment as shown in Figures 3(a) and 3(b).

The pH value of the collected effluent is quiet high (11.6) which indicates the high alkaline nature of the effluent. The chemicals such as sodium hypochlorite, sodium hydroxide, and sodium phosphate used during bleaching and mercerizing process in textile mills are responsible for highly basic nature of effluent [36].  Figure 3(c) indicated that the pH of all cases except 100% was reduced to the acceptable limit as given by EPA [31]. In only 100% textile effluent, a negligible change was observed in pH from 11.63 to 11.29 after 48 h of treatment and remained almost constant. It indicates that growth of macroalgae gets retarded in 100% textile effluent after 48 h as, in 100% textile effluent, toxicity level is quiet high. In 10% and 25% concentrated textile effluent, maximum decrease was in pH which was near control value 7.27 after 120 h.

Thus, a significant reduction in pH of textile effluent was found after phytoremediation process and* C. vulgaris* able to neutralize the alkaline textile effluent. This can be attributed to the fact that the pH reduction might be due to microbial action in anaerobic conditions. During microbial respiration, organic matter decomposes and releases the CO_2_ which may be responsible for decreasing the pH [37]. Also, reduction in pH of waste water is favoured by a consecutive decrease in BOD and COD of effluent [38]. In our studies also, reduction in pH of all batches of phytoremediation experiment was followed by reduction in BOD and COD level with the time of contact. It can also be interpreted from Figures 2 and 3 that the textile effluents of various concentrations (10 to 100%) treated with* C. vulgaris* showed significant variations in all observed parameters within first 24 h of treatment. It indicates that maximum removal of pollutants is obtained within 24 h of treatment with* C. vulgaris*. However, after that, the quality of effluent got improved significantly during 120 h of contact with* C. vulgaris*. It is also interpreted that most of these parameters of treated effluent of the concentration of 10-50% are within the acceptable range given by EPA. The macroalgae* C. vulgaris* used for concentration 10-50% remained in active stage and can be used again for further effluent treatment.

In this research work, reusability of* C. vulgaris* has been also checked for 10%-50% concentrated textile effluent for five runs. For 50% concentrated textile effluent, effective treatment performance has been found for 2 runs only. COD value reduced to 40% in first run and only 15% in second run. For other concentrations of textile effluent, during repeated runs the treatment performance of various parameters reduces with time. For 10% concentration of textile effluent,* C. vulgaris* successfully manage 5 cycles. The COD values reduced to 70%, 56%, 48%, and 32% in 2nd, 3rd, 4th, and 5th, respectively. Hence, these results point out the potential application of* C. vulgaris* plant for tertiary level treatment in ETP of textile effluent.

### 3.2. Correlation Analysis

The correlation matrix for various physiochemical parameters of untreated and treated textile effluent at 24-120 h is given in Table 2. It is clear from Table 2 that all parameters under consideration at different intervals are highly correlated to each other (r > 0.930) except in the case of temperature. As there is only slight change in temperature of waste water while values of other parameters either increase or decrease significantly during the treatment process. Among all the observed parameters, it was found that all parameters have positive correlation between each other except in the case of DO. This result is attributed to the fact that there is an increase in DO values followed by reduction of BOD, COD, pH, TDS, and EC in all concentrations except 100%. It indicates that the* C. vulgaris* absorb textile pollutants which decrease the value of BOD and COD and, as a result, enhances the DO content. As BOD and COD are the most important analytical parameters in assessing effluent pollutants In our study, both BOD and COD showed very strong correlation with each other at different intervals of treatment (r>0.990). Likewise, TDS, EC, and DO also show high correlation with BOD (r > 0.964). A very interesting fact has also been observed that phytoremediation treatment shows the maximum correlation between all important parameters after the time period of 48 h and the trend to remain the same till 120 h.

### 3.3. Analytical Analysis

**Figure 4:**
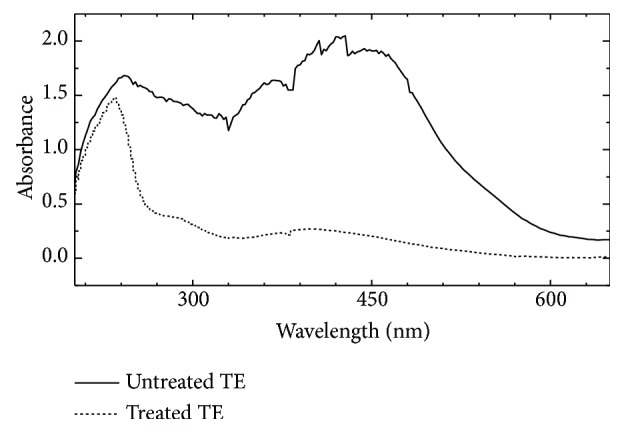
UV-visible spectra of 10% textile effluent before and after treatment of 120 h.

A visible change in colour of textile effluent was observed from dark bluish black to very light colour after 120 h of phytoremediation treatment of effluent in different concentrations. The decolourization efficiency of* C. vulgaris* was observed to be decreased with increase in concentration of effluent. The UV-visible spectra of 10% textile effluent both before and after phytoremediation experiment have been taken (Figure 4).

As textile water sample was highly concentrated; hence spectra were taken at 10% dilution. The spectra which correspond to the components of the textile effluent were presented with wavelengths from 200 to 800 nm. Irregular spectra appeared due to the presence of a number of chemical pollutant impurities of textile effluent. After phytoremediation experiment, the absorbance was found to have dropped from 2.0 to 0.25 and also irregularities disappeared. The results specified potential of* C. vulgaris* towards the removal of pollutants from textile waste water and decolorisation of textile effluent

**Figure 5:**
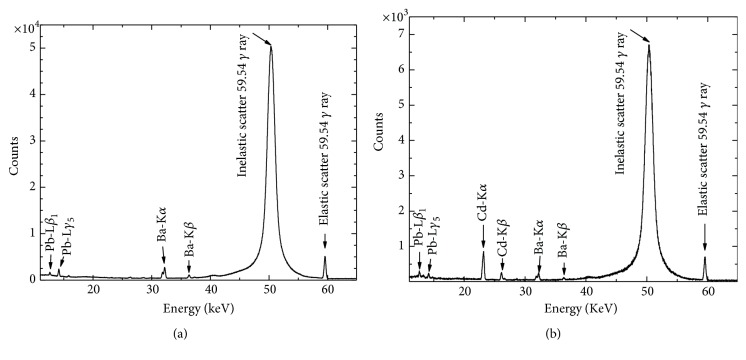
Typical X-ray spectra of* C. vulgaris *(a) before and (b) after treatment with 10% textile effluent.

Typical X-ray spectra of the dried sample of macroalgae* C. vulgaris* before and after treatment with 10% textile effluent is shown in Figures 5(a) and 5(b). The spectra indicate K-X ray peaks of Cd, elastic, and inelastic scattered peak of 59.54 keV and L -X ray peaks of Pb from Pb shielding of detector. The presence of K-X ray of peaks of Cd in the treated spectrum (Figure 5(b)) indicates the extraction of the heavy metal Cd by* C. vulgaris* from textile effluent and reduction in pollution load of textile effluents. Similar study of extraction of heavy metal Cd by* C. vulgaris* from polluted river water was also reported by Laffont-Schwob et al. [32]. The potential applications of* C. vulgaris* for metal removal of effluent have also been reported earlier [38]. These results show the potentiality of* C. vulgaris* to reduce the toxicity level of textile effluent by removing heavy metal.

### 3.4. Phytotoxicity Studies

From Table 3, it is clear that the growth of* Phaseolus mungo* and* Pisum sativum* was totally inhibited in the textile effluent of 50% concentration, while, in case of water and treated effluent, growth was up to mark. The radical and plumule growth of seeds of* Phaseolus mungo* and* Pisum sativum* were observed to be retarded in the textile effluent. The germination growth of both types of seeds was almost the same in water and treated effluent about 95%. This result indicated the removal of toxicity from treated effluent by macroalgae* C. vulgaris*.

Hence, the present investigation showed that* C. vulgaris* have substantial phytoremediation potential for treatment of textile effluents which contain a variety of hazardous chemical constituents and the applicability of aquatic macrophytes* C. vulgaris* for biological treatment of ETP plant of textile waste water at tertiary level. The results of this study help us in developing a reliability simulating model for* in situ* phytoremediation of the textile effluent of various stages by fixing main water quality parameters through submerged aquatic plant.

### 3.5. Reliability Model

**Figure 6:**
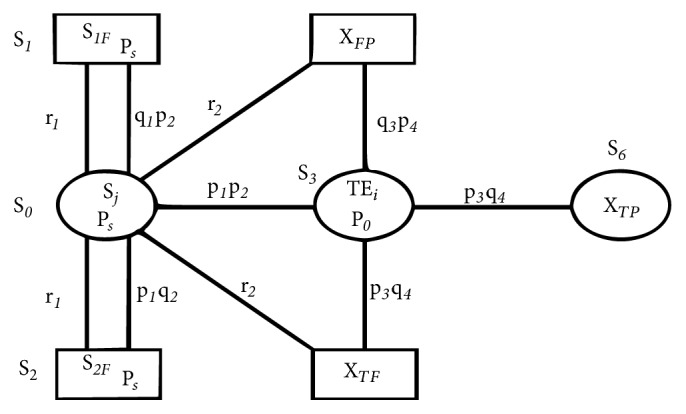
Reliability model for phytoremediation treatment of textile effluent by* C. vulgaris*.

A reliability model is proposed to simulate the treatment of textile waste water through the application of phytoremediation process (Figure 6). The relationship is able to identify percentage level of toxicity tolerance of waste water by macroalgae.

In general, phytoremediation process of treatment of waste water is based on absorption of pollutants present in waste water through a specific type of plant. In turn, it also depends on the concentration of toxic pollutants present in waste water. This helps in determining the potential for macroalgae* C. vulgaris* to absorb pollutants present in specific industrial waste water.

The percentage level of toxicity of waste water is determined in terms of various water quality standards such as pH, BOD, COD, and TDS. Here, we assumed COD as main water quality parameter to access the toxicity level of textile effluent. Hence, a reliability model is given on treating textile waste water with specific macroalgae* C. vulgaris* with the following assumptions:

- A textile waste water system with high pollutant load in which COD is in the range of 100-2000 mg L^−1^ can be treated by phytoremediation process.
- Textile effluent (TE_*i*_) of different concentration with *i* = 1-5 (1-10%; 2-25%; 3-50%; 4-75%; 5-100%) with the optimized amount of macroalgae describes the success of proposed model.
- The volume of textile water under consideration assumed to be fixed; i.e., 5 L.
- Success state is presumed only when specific aquatic macrophytes* C. vulgaris* is used for treatment process as the mechanism of phytoremediation to relate the interaction of pollutants to chemical constituents of the plant.
- Also, the rate of success of process is assumed to be varied from variation in amounts of macroalgae.
- Other parameters such as temperature, humidity, and pressure were presumed to be constant.

The main input and output terms which describe success and failure of the model are provided in Table 4.

Initially, it is described in the model that probability of success of phytoremediation system exists only if COD of textile is less than 2000 mg L^−1^ [39]. Hence, it is necessary to evaluate water quality parameter COD to proceed the phytoremediation of probabilities P1 and P2. Further, the probability of the success rate of the process can be achieved only with P3 and P4. Hence, only integration of P1 with P2 and P3 with P4 leads to the success of the model. The system is said to be in failure state in two conditions, namely, either textile effluent concentration or amount of algae are not appropriate. Two replacement policies r1 and r2 have also been introduced, i.e., one at an initial stage and the other at the time of phytoremediation process. The replacement cost at treatment stage is much more than at initial stage.

## 4. Conclusion

Phytoremediation experiments have been carried out for the treatment of textile effluent using an aquatic macroalgae* C. vulgaris*. The results obtained in this research indicated the phytoremediation potential of* C. vulgaris* for reducing toxicity of textile effluent.* C. vulgaris* efficiently reduced BOD, COD, pH, EC, and TDS of the 10-50% of concentrated textile effluent within 120 h of treatment. The DO content has been increased with increase in photosynthetic activity. In 10% and 25% concentrated textile effluent, maximum decrease was in pH which was near control value 7.27 which indicate the potential of macroalgae to regulate pH.* C. vulgaris* successfully managed to treat the textile effluent up to 5 runs for 10% concentration. Thus, for enhancing purification process of the textile effluents,* C. vulgaris* is highly recommended for tertiary level water treatment in ETP plants of textile industry.* C. vulgaris* would also be able to handle the polluted water in which exceeded emission standards pollutants get discharged. The phytotoxicity studies support the improvement of water quality after treatment. The absorption of toxic heavy metal Cd along with reduction in BOD and COD by* C. vulgaris *highlights that the treatment of textile effluent by C. vulgaris as more effective solution. Hence,* C. vulgaris *potential can be utilized for phytoremediation of water resources contaminated with textile effluent.

## Figures and Tables

**Table 1:** Comparison of physiochemical parameters of untreated effluent (100%) and 10% treated effluent before and after treatment of 120 h with EPA discharge limit.

Parameter	EPA limit	Untreated effluent (100%)	Untreated effluent (10%)	Treated effluent (10%)
pH	6-9	11.63	8.86	7.30

Temperature (°C)	40	46	33	35

DO (mg L^−1^)	-* *-* *-	0.6	5.6	7.2

BOD(mg L^−1^)	50	395	65.50	14.41

COD (mg L^−1^)	80	1926	216.29	38.93

TDS (mg L^−1^)	2000	4210	525	168

EC (mho cm^−1^)	1.5	3.87	0.75	0.07

**Table 2:** Correlation matrix between different physiochemical parameters at different time interval.

	Conc.	pH	TDS	DO	BOD	COD	EC	Temp
		**Untreated (0 h)**						
Conc.	1.000							
pH	0.959	1.000						
TDS	0.999	0.945	1.000					
DO	-0.959	-0.999	-0.944	1.000				
BOD	0.998	0.973	0.995	-0.973	1.000			
COD	0.999	0.945	1.000	-0.945	0.995	1.000		
EC	0.996	0.970	0.993	-0.968	0.996	0.993	1.000	
Temp	0.558	0.623	0.551	-0.588	0.569	0.542	0.611	1.000
		**Treated (for 24 h)**						
Conc.	1.000							
pH	0.996	1.000						
TDS	0.992	0.996	1.000					
DO	-0.975	-0.964	-0.942	1.000				
BOD	0.995	0.996	0.992	-0.964	1.000			
COD	0.988	0.990	0.997	-0.931	0.992	1.000		
EC	0.994	0.988	0.983	-0.969	0.996	0.987	1.000	
Temp	0.558	0.563	0.536	-0.582	0.623	0.571	0.637	1.000
		**Treated (for 48 h)**						
Conc.	1.000							
pH	0.985	1.000						
TDS	0.243	0.251	1.000					
DO	-0.041	-0.061	-0.974	1.000				
BOD	0.282	0.302	0.978	-0.961	1.000			
COD	0.983	0.977	0.094	0.104	0.152	1.000		
EC	0.974	0.987	0.399	-0.213	0.446	0.944	1.000	
Temp	0.0734	0.197	0.530	-0.577	0.554	0.003	0.248	1.000
**Treated (for 72 h)**								
Conc.	1.000							
pH	0.988	1.000						
TDS	0.987	0.987	1.000					
DO	-0.989	-0.988	-0.986	1.000				
BOD	0.982	0.997	0.977	-0.975	1.000			
COD	0.983	0.991	0.975	-0.967	0.997	1.000		
EC	0.978	0.985	0.994	-0.985	0.971	0.962	1.000	
Temp	0.073	0.130	0.120	-0.213	0.071	0.005	0.184	1.000
**Treated (for 96 h)**								
Conc.	1.000							
pH	0.977	1.000						
TDS	0.987	0.974	1.000					
DO	-0.986	-0.976	-0.993	1.000				
BOD	0.973	0.999	0.964	-0.970	1.000			
COD	0.980	0.999	0.975	-0.975	0.997	1.000		
EC	0.967	0.968	0.994	-0.989	0.958	0.967	1.000	
Temp	0.073	0.033	0.108	-0.192	0.0199	0.005	0.150	1.000
**Treated (for 120 h)**								
Conc.	1.000							
pH	0.995	1.000						
TDS	0.986	0.984	1.000					
DO	-0.983	-0.971	-0.993	1.000				
BOD	0.965	0.938	0.952	-0.967	1.000			
COD	0.979	0.959	0.975	-0.984	0.995	1.000		
EC	0.965	0.959	0.993	-0.991	0.943	0.966	1.000	
Temp	0.074	0.066	0.114	-0.161	-0.0250	0.005	0.150	1.000

**Table 3:** Comparison of phytotoxicity of 50% untreated and treated textile effluent with control after 10 days.

Parameters	*Phaseolus mungo*			*Pisum sativum*		
	Textile Effluent			Textile Effluent		
	Control	Untreated	Treated	Control	Untreated	Treated
G (%)*∗*	100	0	90	100	0	80
PL*∗* (cm)	7.1	0	5.6	10.6	0	7.2
RL *∗*(cm)	3.7	0	2.0	5.6	0	4.1

*∗* G (%), germination percentage; PL, plumule length; RL, radical length.

**Table 4:** Nomenclature for reliability mathematical model.

S_*0*_, S_*3*_, S_*6*_:	Operative state of Phytoremediation process.
S_*1*_, S_*2*_, S_*4*_, S_*5*_:	Failure state of Phytoremediation process.
S*j*:	Water Quality Parameter COD range 100-2000 mg L^−1^
P*s*:	*C. vulgaris* is in unreactive mode (standby mode)
P*o*:	*C. vulgaris* is in reactive mode.
S_*1F*_:	Failure due to low range of *j*.
S_*2F*_:	Failure due to high range of *j*.
TE_*i*_:	Textile Effluent in different concentration
	where *i* = 1-5(10%, 25%, 50%,75%,100%).
P_*1*_:	Probability of *j *≥ lower range of COD.
P_*2*_:	Probability of *j* ≤ upper range of COD
P_*3*_:	Probability of TE_*i*_ in required concentration when *i* = 1-3.
P_*4*_:	Probability in 500 mg L^−1^* C. vulgaris* required amount.
q_*1*_:	Failure Probability of *i* not greater than lower range of parameters.
q_2_:	Failure Probability of *i* not less than upper range of parameters.
q_*3*_:	Failure Probability of TE_*i*_ of concentration when *i* = 4 & 5.
q_*4*_:	Failure probability of *C. vulgaris* amount less than 500 mg.
X_*TP*_:	Success state of treatment of TE.
X_*TF*_:	Failure state due to TE of concentration *i* = 4 & 5.
X_*FP*_:	Failure state due to algae with less amount.
r_*1*_:	Replacement cost due to failure in initial stage (S_*1F*_ and S_*2F*_).
r_*2*_:	Replacement cost due to failure in treatment stage (X_*TF*_ and X_*FP*_).

## Data Availability

The data used to support the findings of this study are available from the corresponding author upon request.

## Abbreviations

EPA: Environmental Protection Agency
APHA: American Public Health Association
BOD: Biological Oxygen Demand
COD: Chemical Oxygen Demand
DO: Dissolved Oxygen
TDS: Total Dissolved Solids
EC: Electrical Conductivity
EDXRF: Energy Dispersive X-Ray Fluorescence.
